# Development of Nutraceutical Ice Creams Using Flour Yellow Worm Larvae (*Tenebrio molitor*), Chia (*Salvia hispanica*), and Quinoa (*Chenopodium quinoa*)

**DOI:** 10.3389/fvets.2021.629180

**Published:** 2021-12-07

**Authors:** Ana Gabriela Hernández Toxqui, Jazmín Ramírez Ramírez, José Manuel Pino Moreno, José Moisés Talamantes Gómez, Sergio C. Angeles Campos, Juan Carlos Ramírez Orejel

**Affiliations:** ^1^Food and Biotechnology Department, Faculty of Chemistry, National Autonomous University of Mexico, Mexico City, Mexico; ^2^Institute of Biology, National Autonomous University of Mexico, Mexico City, Mexico; ^3^Animal Nutrition and Biochemistry Zoology Department, Faculty of Veterinary Medicine and Animal Husbandry, National Autonomous University of Mexico, Mexico City, Mexico

**Keywords:** edible insects, *Tenebrio*, larvae, nutritional value, functional foodstuff

## Abstract

Functional ice creams were developed by adding larvae of the insect *Tenebrio molitor* mixed with a seed (*Salvia hispanica*) and a pseudocereal (*Chenopodium quinoa*) to strawberry–cranberry ice cream. The objective was to increase micronutrients, macronutrients, and antioxidants, thus rendering the product a food complement. Four ice cream formulations were manufactured: the control strawberry–cranberry ice cream and three experimental mixtures, one of them with an addition of *Tenebrio* larvae (HT) and two others with a combination of *Tenebrio* larvae, chia (HTC), and quinoa (HTQ). The ice creams were submitted to proximate chemical analysis: mineral, fatty acid, vitamin, and one antioxidant (cyanidin 3 glucoside) determination. The strawberry–cranberry ice cream was used as a control formulation to evaluate if there were significant differences among nutrients, to which a Dunnett test with a critical value of α = 0.05% was applied. The three formulations that were studied showed a significant increase in the analyzed micronutrients and macronutrients compared to the control formulation. We observed increases of up to 62% in lipid content in the HTC formulation, while an increase of 41% in the protein content of the HT formulation was observed. We quantified an increase and enrichment of vitamins and minerals in the manufactured products, so that their nutritional value was significantly enhanced. In the determination of cyanidin 3 glucoside, we found that the formulation to which chia had been added showed an increase of 74% as compared to the control ice cream; this is important because anthocyanins are a group of flavonoids that stand out for their antioxidant and antimutagenic capabilities.

## Introduction

Nutrition relies on different factors, such as place of residence, surrounding habitat, available alimentary resources, religion, ethnic group, education, migration, colonization, trade, and the ways in which food is cooked or preserved and stored, which have their origins in ethnic footprints (1). Besides, in the last decades, there has been an increasing interest among some population sectors not only in favoring a sustainable and healthy diet but also in learning the nutritional value of foodstuffs and their effects on the human body. In the industrialized world, the concepts in nutrition are changing significantly. From a former emphasis on survival, through hunger satisfaction and, more recently, food safety, food sciences now aim at developing foods to promote well-being and health while reducing the risk of some major diseases (2). It is because of this that the food industry currently faces the challenge of developing functional foodstuffs that respond to consumer tastes, needs, and demands (3) as well as their need for learning the nutritional function of the ingredients used in their manufacture (4).

In this context, the search for functional foodstuffs appears to be urgent. Bello (5) defines a functional product as “any product that can be considered as a foodstuff, or part of a foodstuff, that is capable of providing health benefits, includ[ing] disease prevention and treatment.” The benefits could be either maintenance or promotion of a state of well-being or health and a reduction of the risk of a pathologic process or a disease. Among functional foodstuffs, different compounds of macronutrients and micronutrients can be found, such as carbohydrates, fats, amino acids, minerals, and vitamins.

Anthropoentomophagy refers to the consumption of edible insects. It is the human consumption of insects, an activity that has been practiced since the rise of mankind in countries such as China and Mexico, whereas in other countries—particularly in Europe—it has recently been adopted and valued (6). The importance of the role of insects as functional foodstuffs has been quantified in some respects. For example, several chemical compounds have been quantified, such as pigments, sterols, coumarins, terpenoids, iridoids, prostaglandins, pterines, alkaloids, and quinines (7–9). Because of this, insects are an important source of these compounds in the research carried out inside the alimentary industry. Nonetheless, they have received scarce attention in the scientific arena (10). For the reasons aforementioned, we considered of the manufacture of functional ice creams using the larvae of *Tenebrio molitor*, the mealworm, as the protein ingredient of interest, in which the presence of good-quality nutrients has been demonstrated (11) and also because insects represent a source of functional compounds yet to be discovered (9).

It is important to point out that due to the current demand for new sources of protein for human and animal nutrition, the insect *T. molitor* has been analyzed quite thoroughly to assess its nutritional value; thus, the state of the art in this respect is very detailed: for instance, we know that the larvae harbor 53.13% protein, 36.65% fat, 3.19% total ash, 5.10% crude fiber, and 1.90% nitrogen-free extract (12).

Ravzanaadii et al. (13) published an excellent research paper that allows us to explain this aspect in a brief way: *T. molitor* larvae, adults, exuviae, and excreta contained 46.44, 63.34, 32.87, and 18.51% protein, respectively, and Aguilar-Miranda et al. (14) reported higher protein content (58.4% for larvae).

This protein was rich in amino acids such as isoleucine, leucine, and lysine. The results showed that amino acid composition met the requirements of not only domestic animals but also human beings: cysteine + methionine (1.18 g/100 g protein) and phenylalanine + tyrosine (5.21 g/100 g protein). Overall, insects contain higher amounts of lysine and threonine, but lower amounts of methionine/cysteine (15).

The total fat content was 32.7, 7.59, 3.59, and 1.3% for *T. molitor* larvae, adults, exuviae, and excreta, respectively. The mealworm larvae presented an average fat content of 32.7%, a quantity that is greater than that of locusts (21.5%) and grasshoppers (3.8%) but less than that of termites (61.1%) (16).

In fatty acid composition, they have a high component of oleic acid (C18:1), linoleic acid (C18:2), and palmitic acid (C16) in larvae, with values of 43.17, 30.23, and 16.72%, respectively. Also, the amount of ω-6 was determined to be significant.

These essential fatty acids demonstrate that insects can be used for many other purposes such as feeding of domestic animals and as food supplements for human beings. Besides, it has been reported that insects' caloric value is 50% higher than that of soybeans, corn, and beef (15). Considerably high amounts of unsaturated fatty acids in larvae, adult, exuviae, and excreta have a similar composition to those of poultry and fish.

Furthermore, larvae have among their fats lauric acid, myristic acid, palmitic acid, palmitoleic acid, stearic acid, oleic acid, and linoleic acid (17).

In mineral composition, as in other insects, mealworm contained poor sources of calcium which were 434.59, 484.39, 801.14, and 1,537.97 mg/kg for larvae, adult, exuviae, and excreta, respectively. Even when the calcium content is considerably low in mealworm, it contains instead high levels of P (18).

Therefore, in terms of nutritional content, edible insects (grasshoppers, crickets, termites, ants, beetle larvae, moth caterpillars, and pupae) have been stated to have more of it when compared to other conventional foods.

There will be a huge economical change involved if insects become more commonly considered as an acceptable food source for both human beings and domestic livestock in industrial countries (15).

Insects have potentially been used as a human nutrient source in traditional food among indigenous people throughout history (15). Insects represent 5–10% of animal protein source and also supply fats, calories, vitamins, and minerals among some ethnic groups (12, 19). Furthermore, as an attractive and important natural food source, insects have been used by various kinds of animals, such as birds, lizards, snakes, amphibians, fish, insectivores, and other mammals (20). They are normally encouraged to be sold alive, yet they are likewise sold canned, dried, or powdered; powdered larva is a high-grade item considered as an enhancement to conventional foods (21).

The production and innovation of food based on insects have become a trend in recent years, and in many parts of the world, it is something completely normal; such is the case in Germany, Belgium, Brussels, Costa Rica, Spain, and Holland, among others. In the Netherlands and China, insects are used as food ingredients and are progressively viewed as an option for meat (22).

Insects have an excellent nutritional value, and their flavor is also fabulous; that is to say, they are even a delicacy; mealworm is widely used in various types of foods, for example, with the larvae, ice creams were prepared at the insect experience festival in Wageningen, The Netherlands (23).

Also, with *Tenebrio*, van Huis et al. (23) reported a wide variety of recipes among which include “triangles,” bitterbug bites, bugsitgoreng, nutty mealworms, quick meatballs, flower power salad, pumpkin soup, minestrone, tagliatelle with creamy herb sauce, ravioli, hakunamatata, chukli con carne, chop suey, jambalaya, insect burgers, vol-au-vent, quiche, chebugschichiu, pizza, buglasva, and tarte tatin.

For the aforementioned reasons, in this aspect, it is clear that the future of food lies in insects and in the innovation of new, healthy, nutritious, and delicious products.

As regards ice cream and its importance, we can begin by pointing out that ice cream is defined by its composition and structure: it is a frozen foodstuff that, in general, is manufactured using dairy products such as milk or cream mixed with fruit or other ingredients, along with sugar, flavorings, sweeteners or honey, and stabilizing substances (24). To manufacture high-quality ice cream, it is essential to have excellent ingredients and a mixture that is formulated and well-balanced to provide an adequate function between each ingredient in order to obtain organoleptic properties that may be to the liking of the consumers. All of this is fundamental for the physicochemical properties of the product. On the other hand, ice cream has been considered as a dessert, that is, with minimal nutritional value. However, it has recently been reported that milk and fruit-based ice cream has a fundamental alimentary value (25).

As to the general characteristics of the ingredients, the nutritional value of edible insects in Mexico has been widely studied. Edible insects have been the source of 10–81% of protein (26); essential and non-essential amino acids, for instance, leucine (5.20–8.46) (g/100 g of protein) (27); vitamins, especially the ones from group B such as thiamine (0.08–6.110) and riboflavin (0.050–3.230) (mg/100 g^−1^) (28); and minerals such as sodium (0.020–1.608), potassium (0.014–2.912), and calcium (0.040–0.224) (g/100 g) (29).

In certain cases, they are rich in fats (12, 17); for example, the beetle known as *T. molitor* has been used as a source of protein with different alimentary purposes throughout the world (30). The larvae harbor up to 58 g of protein, for every 100 g of dry weight, and 38.29 g of fats with a high content of unsaturated fatty acids (12).

*Chenopodium quinoa* is an annual plant with a height between 1 and 2 whose seeds have a 2-mm diameter. These seeds can be white, red, or black and are edible when consumed whole, having a high nutritional value (31). Even the FAO (32) has furthered it as a crop with the potential to guarantee alimentary food security in the 21st century (33).

*Salvia hispanica* is an herbaceous, annual plant with a height of 1 or 1.5 m whose seeds measure a mean of 2-mm length and of 1.5-mm width (34). The oleaginous seed of this plant has a significant quantity of ω-3 fatty acids (α-linolenic acid), fiber (+30%), high-biological-value proteins, and natural antioxidants. It also has essential and non-essential amino acids, vitamins, minerals, and dietary fiber (35).

An antioxidant is defined as a substance that is found in foodstuffs in our everyday life and that can prevent the adverse effects of reactive species on the normal physiological functions of human beings; they also delay the aging process (36). Quinoa seeds possess a high antioxidant activity as they contain anthocyanins (37). Given the aforementioned facts, the objective of this research was to manufacture functional ice creams with both animal and plant products so as to enhance their nutritional (proteins, fatty acids, vitamins, minerals, and antioxidants) value through the use of non-conventional ingredients such as the larvae of *T. molitor*, chia, and quinoa.

## Materials and Methods

This research comprised three phases.

The first one consisted of (a) the purchase of the ingredients for the ice cream foundation: water (Bonafont, Mexico City), pasteurized cow's milk cream, powdered whole milk (Cuautitlan Izcalli, State of Mexico), sugar (Trademark Zulka, Monte Caucaso 915, Lomas de Chapultepec, Miguel Hidalgo, C.P. 11100 Mexico City, México), gums (carob bean, carboxymethyl cellulose, and carrageenan) (Cosmopolitan Drugstore, Avenida Revolución 1080 Col. Mixcoac, Benito Juárez, Mexico City, Mexico), and the raw materials to provide it with flavor and functionality (strawberry, cranberry, chia and quinoa seeds); the ice cream foundation was then left to mature for 24 h at 4°C; and (b) the selection and cleansing of the insect larvae: these were obtained from the Institute of Biology, UNAM, National Autonomous University of Mexico, where they are cultured in controlled conditions at a temperature of 26–27°C and a relative humidity of 60%, being fed on wheat bran for a period of 90 days; the larvae were dipped in boiling water for 5 min—this was done in order to avoid them changing color during the procedure—and afterwards they were stored in freezers at −4°C; and (c) quinoa's preparation: seed conditioning achieved by boiling it for 10 min and drying it in a stove at 80°C for 6 h.

In the second phase, we developed four formulations for strawberry–cranberry ice cream: HCOo, which was used as a control; HT with *T. molitor* larvae; HTC, larvae mixed with chia; and HTQ, larvae mixed with quinoa. These were manufactured according to the specifications contained in NMX-F-714-COFOCALEC-2012 (38). Three control batches of ice cream HCOo of 1 kg each were made; the same was done for the experimental formulations HT, HTC, and HTQ; all the formulations were prepared on the same day and in triplicate.

Also, we chose an experimental design to determine if there existed significant differences among the experimental ice cream formulations (as a whole, not by their components) and the conventional ice cream (control ice cream) whose basic formulation was used to prepare all the ice creams, as well as to verify if there was an increase in the nutrients when these were assessed by means of diverse types of chemical analysis.

The substitution and inclusion of the seed (chia), pseudocereal (quinoa), and *Tenebrio* larvae ingredients were done with respect to the control formulation to observe how the nutrients varied and to verify that the objective of the investigation was achieved.

In this case, we considered the amount of matured ice cream base (for the preparation of that quantity, the percentages expressed in the table cover the 500 g of matured ice cream base, to make 1 kg of total ice cream) and the adjustment of solids with respect to the NMX-F-714-COFOCALEC-2014 standard to consider it milk ice cream.

In the third phase, we carried out the chemical analyses to elicit the chemical composition of each of the ice creams: (a) proximate chemical analyses (fats, protein, dietary fiber, reducing carbohydrates, ash, and carbohydrates), (b) liposoluble and hydrosoluble vitamin determination, (c) fatty acid quantification, and (d) assessment of the antioxidant known as cyanidin chloride. Strawberries, cranberries, chia, and quinoa were not analyzed; nevertheless, for chia and quinoa, we used the data from several reliable bibliographic sources published in the United States or even by the FAO (39). The larvae of *T. molitor* were in fact analyzed.

The general process of the ice cream manufacture was as follows. The ingredients were weighed, mixed, and pasteurized at 68°C for 30 min. The addition of the strawberry–cranberry pulp, seeds, and larvae compromised the aging of the foundation at 4°C/24 h, which was then whipped for 10 min and frozen at −4°C.

To carry out chemical determinations, official published and standardized methods were used: for humidity (40), we determined humidity of foodstuffs by thermal treatment (the sand or gauze method); by means of the AOAC (41) procedures, we quantified fat in ice cream and frozen desserts (952.06), protein in ice cream and frozen desserts (930.33), total dietary fiber in foods (985.29); by the enzymatic-gravimetric method, we determined ash of milk (945.46); by proximate analysis of milk-based infant formulation, we determined carbohydrates (986.25); and finally, to determine reducing carbohydrates, we used the 3,5-dinitrosalicylic method.

The methods employed for the quantification of micronutrients in the manufactured formulations were the following. The mineral profile was quantified by the AOAC 985.3 method that assesses minerals in ready-to-feed milk-based infant formulations by atomic absorption spectrophotometry (Perkin Elmer Model 3110 Waltham, Massachusetts, USA). For the fatty acid profile, we applied the gas chromatography method GC-FID (Perkin Elmer Model Auto System XL Waltham, Massachusetts USA) (AOAC 969.33).

To assess the liposoluble vitamin profile, the method of Chotyakul et al. (42) was used. For identification and quantification, we used a vitamin A, E, D, and K stock solution with a concentration of 500 μg/ml. The calibration curve was constructed from the mix in which the vitamins ranged in an interval of 50–500 μg/ml. The vitamin composition analysis of the formulations was carried out by means of chromatogram interpretation.

To quantify hydrosoluble vitamins, we used the method from Gliszczynska and Rybicka (43) and Rokayya et al. (44). For identification and quantification, we used a vitamin B1, B2, B3, B6, B9, and B12 stock solution with a concentration of 500 μg/ml. The calibration curve was constructed from the mix in which the vitamins ranged in an interval of 50–500 μg/ml.

For the mineral profile, we used AOAC 985.35. And for cyanidin quantification, we applied a modified method from Martínez-Cruz et al. (45); we interpreted the chromatograms and constructed the corresponding calibration curves.

To evaluate if there were significant statistical differences between the nutrients thus determined, we carried out a one-factor analysis of variance (ANOVA) between the four formulations, as well as a Dunnett test to identify which macronutrients or micronutrients show variations with respect to the control ice cream. The Dunnett test is used to create confidence intervals between the mean of each factor level and the mean of the control group. In such a way, this test compares each formulation finding if there are any or no differences between each of them, but also with respect to the control with a confidence level of 0.05 (46).

We must initially point out that all the results presented in our tables are the mean obtained from three repetitions (that mean had a standard deviation and a variation coefficient or error lower than 5%) and that the values in the rows with different letters signal that between them there exists a significant difference; only the values of carbohydrates were obtained by difference, and in each table, the highest quantified values are indicated in bold.

## Results and Discussion

### Proximate Chemical Analysis

According to NMX-F-714-COFOCALEC-2012 (38), the products we manufactured were classified as “milk ice cream” as they comply with the minimal physicochemical specifications required. In Table 1, the results of the proximate chemical analysis are presented, upon a dry basis of g/100 g of dry sample, since it is human food and it is reported in accordance with the labeling regulations in force in Mexico, NOM-051-SCFI/SSA1-2010 (47).

**Table 1:** Results of the proximate chemical analyses of the ice cream upon a dry basis (g/100 g of sample).

**Sample**	^*^HCOo	**HT**	**HTC**	**HTQ**
Fat	11.07^a^ ± 0.46	14.34^b^ ± 0.10	**15.55**^**b**^ ± 0.16	12.01^b^ ± 0.08
Protein	9.36^a^ ± 0.25	**11.81**^**b**^ ± 0.25	11.36^b^ ± 0.26	10.95^b^ ± 0.25
Dietary fiber	15.01^a^ ± 0.93	13.89^a^ ± 0.51	**21.22**^**b**^ ± 0.69	15.59^a^ ± 0.34
Reducing carbohydrates	26.91^a^ ± 0.61	25.87^b^ ± 0.26	**27.09**^**a**^ ± 0.14	26.76^a^ ± 0.28
Ash	1.88^a^ ± 0.05	2.11^b^ ± 0.08	**2.24**^**b**^ ± 0.00	2.02^a^ ± 0.07
Carbohydrates	**35.77** ± 0.00	31.98 ± 0.00	22.54 ± 0.00	32.67 ± 0.00

* *HCOo, control ice cream; HT, Tenebrio molitor ice cream; HTC, Tenebrio and chia ice cream; HTQ, Tenebrio and quinoa ice cream*.

*a,b *The values in the rows with a different letter indicate that there exists a significant difference among them. Coefficient of variation (CV), known as relative standard deviation (RSD), corresponds to the average value of the three samples processed, that is, less than 3% and with an alpha level of significance equal to 0.05 (α = 0.05). The highest values quantified in each determination made in bold*.

We observed significant differences in composition between the control ice cream and the functional formulations: these are due to the addition of chia, quinoa, and the insect larvae. In this case, the fiber content corresponds to the formulations of elaborate ice cream, that is, to the total mixture of all the ingredients. Consequently, it does not refer to *Tenebrio*. In the same table, reducing carbohydrates or reducing sugars are defined as sugars that can be oxidized by weak oxidizing agents; for example, all monosaccharides are reducing sugars.

### Lipids

The experimental samples (HT, HTC, and HTQ) have significant differences in their lipid contents that enhance their nutritional value and consistency, with the HTC formulation being the one that shows the highest proportion by the addition of such ingredients. Ramos-Elorduy et al. (11) mentioned that the larvae possess a lipid content of 38.29%, and chia, according to the USDA (48), is a seed with a high lipid content of 30.74% upon a dry basis. In this case, the FAO (49) recommends that adults consume a minimum of 15% of their calories as lipids (33 g of lipids for a 2,000 kcal/day diet) and a maximum of 35% (78 g of lipids for a 2,000 kcal/day diet) to avoid disease.

As Table 1 shows, no ice cream provides more than 9% of what is recommended by the FAO (49); that is, ice cream ingestion could complement the diet so as to reach the suggested fat percentage. When the types of lipids found in ice cream were analyzed, it was observed in Table 2 that although 65–70% of the total lipids were saturated, no formulation surpassed the energy of 10% (22 g of saturated lipids for a 2,000 kcal/day diet) that the FAO (49) defines as the maximum limit. Furthermore, all of the experimental formulations provided a significant contribution of unsaturated fatty acids thanks to its ingredients.

**Table 2:** Results of the types of fatty acids in each formulation manufactured (g FA/100 g of fresh sample).

**Fatty acids**	**HCOo**	**HT**	**HTC**	**HTQ**
*Butyric C4:0*	3.73^*^ ± 0.00	3.15^b^ ± 0.00	2.86^b^ ± 0.00	2.37^b^ ± 0.03
*Caproic C6:0*	1.21^a^ ± 0.00	0.62^b^ ± 0.01	**1.22**^**a**^ ± 0.02	0.88^b^ ± 0.00
*Caprylic C8:0*	**1.43**^**a**^ ± 0.01	0.77^b^ ± 0.02	1.37^b^ ± 0.02	0.97^b^ ± 0.00
*Capric C10:0*	2.61^a^ ± 0.02	2.43^b^ ± 0.01	**2.77**^**b**^ ± 0.03	2.16^b^ ± 0.01
*Lauric C12:0*	4.36^a^ ± 0.01	4.06^b^ ± 0.01	3.88^b^ ± 0.03	**3.76**^**b**^ ± 0.05
*Tridecanoic C13:0*	**0.91** ± 0.00	0.13 ± 0.00	—	—
*Myristic C14:0*	**11.23**^**a**^ ± 0.05	10.75^b^ ± 0.00	8.89^b^ ± 0.03	10.52^b^ ± 0.1
*Myristoleic C14:1*	**1.49**^**a**^ ± 0.00	0.96^b^ ± 0.00	1.35^b^ ± 0.04	1.01^b^ ± 0.01
*Pentadecanoic C15:0*	**1.67**^**a**^ ± 0.02	1.03^b^ ± 0.00	1.33^b^ ± 0.02	1.39^b^ ± 0.02
*Palmitic C16:0*	29.29^a^ ± 0.19	**32.96**^**b**^ ± 0.77	30.66^b^ ± 0.02	31.96^b^ ± 0.51
*Palmitoleic C16:1n7*	1.59^a^ ± 0.02	1.41^b^ ± 0.00	**1.72**^**b**^ ± 0.07	1.53^a^ ± 0.01
*Heptadecanoic C17:0*	**0.83**^**a**^ ± 0.01	0.43^b^ ± 0.00	0.72^b^ ± 0.01	0.61^b^ ± 0.00
*Stearic C18:0*	10.60^a^ ± 0.32	**10.91**^**a**^ ± 0.10	10.03^b^ ± 0.08	10.64^a^ ± 0.12
*Oleic C18:1n9*	22.00^a^ ± 0.17	**25.30**^**b**^ ± 0.61	23.61^b^ ± 0.03	23.59^b^ ± 0.17
*Linoleic C18:2n6*	2.89^a^ ± 0.02	3.37^b^ ± 0.01	**3.80**^**b**^ ± 0.01	**4.95**^**b**^ ± 0.02
*γ-Linolenic C18:3n6*	—	—	0.28 ± 0.01	—
*α-Linolenic C18:3n3*	0.35^a^ ± 0.00	0.06^b^ ± 0.00	**1.78**^**b**^ ± 0.00	0.21^b^ ± 0.00
*Arachidonic C20:0*	**2.36**^**a**^ ± 0.01	1.49^b^ ± 0.09	2.20^b^ ± 0.01	2.22^b^ ± 0.01
*Eicosenoic C20:1*	**0.98**^**a**^ ± 0.02	0.09^b^ ± 0.01	0.82^b^ ± 0.00	0.717^b^ ± 0.00
*Behenic C22:0 and Erucic C22:1*	**0.78**^**a**^ ± 0.00	—	0.66^b^ ± 0.00	0.458^b^ ± 0.00
Total Lipids	4.25	5.427	**6.924**	4.943
Saturated^**^	2.977	3.733	**4.567**	3.337
Unsaturated^***^	1.275	1.694	**2.357**	1.606
Monounsaturated	1.137	1.508	**1.950**	1.351
Polyunsaturated	0.138	0.187	**0.407**	0.255

*HCOo, control ice cream; HT, Tenebrio molitor ice cream; HTC, Tenebrio and chia ice cream; HTQ, Tenebrio and quinoa ice cream*.

* *The values in the rows with a different letter indicate that there exists a significant difference among them. Coefficient of variation (CV), known as relative standard deviation (RSD), corresponds to the average value of the three samples processed, that is, <3% and with an alpha level of significance equal to 0.05 (α = 0.05)*.

*Saturated Fatty Acid*

** ∑*SFA = (C4:0 + C6:0 + C8:0 + C10:0 + C12:0 + C13:0 + C14:0 + C15:0 + C16:0 + C17:0 + C18:0 + C20:0 + C22:0). Unsaturated Fatty Acid classification*

*** ∑*MUFA = (C14:1 + C16:1 + C18:1 + C20:1 + C22:1). ∑PUFA = (C18:2 + C18:3n6 + C18:3n3). The highest values quantified in each determination made in bold*.

When carrying out the study, the lipids quantified by gas chromatography are classified as saturated or unsaturated, and these are simultaneously reclassified into monounsaturated and polyunsaturated; they are added, and these are the values reported in Table 2.

In Table 2, we also show the results of the type of fatty acids found in each formulation (g FA/100 g of fresh sample). The experimental formulations have a different profile to that of the control ice cream, but we must point out that the addition of *T. molitor*, chia, and quinoa—being ingredients with high levels of unsaturated fatty acids—diminished the percentage of the saturated ones. The predominant fatty acids in the ice creams were palmitic acid, which varied between 29 and 32% and was the most abundant fatty acid in the HT and HTC formulations, and oleic acid, which ranged from 22 to 25% and was the only unsaturated acid present in important quantities in the experimental formulations.

The latter is due to the fact that the foundation to manufacture the ice cream is made up of dairy products in which saturated fats make up 70% of the total fat weight, with palmitic acid being the most common (50) in the results of the fatty acid profile of the formulations. On the other hand, milk lipids are very poor in polyunsaturated fatty acids (linoleic and α-linolenic) (51). That is, the control ice cream has a very similar lipid profile to that of milk, so when quinoa, chia, and larvae are added, a significant increase was shown by linoleic acid (C18:2ω-6); this is why it is important to include it in the diet. Table 2 also shows that the HT formulation was characterized by having a very favorable proportion of ω-6/ω-3 fatty acids. This composition can be considered an indicator of its high nutritional value (52).

The adequate proportion of these fatty acids, ω-6/ω-3, can be considered as another determining factor of the high quality and nutritional value maintained in the investigated formulations (52); for example, ω-3 and ω-6 help to reduce LDL cholesterol in blood, and AGP ω-3 reduces triglyceride levels and platelet aggregation and promotes the immune response. The greatest beneficial effect of this type of polyunsaturated fatty acids resides in their antiarrhythmic mechanism that improves cardiovascular diseases. In addition, recent studies have suggested that they also play a fundamental role in reducing the risks derived from diseases such as type 2 diabetes or hypertension (53).

Table 1 shows the proportions of protein, carbohydrates, reducing carbohydrates, dietary fiber, and ash.

### Protein

This is a macronutrient, and the ice cream with added chia and quinoa showed significant change with respect to HCOo. Nevertheless, the addition of larvae to the formulations had a positive impact, as it increased its content due to the fact that larvae have a high protein content (58%). When combining larvae with the seed or pseudocereal, a decrease was seen in the protein percentage, as these last two ingredients provided a higher quantity of dietary fiber, fat, and/or carbohydrates. All of the manufactured ice creams (except HCOo, 9.36%) had a greater protein content than commercial ice cream. Our manufactured ice cream provided 7% to 10% of the protein required daily, so it could be considered a good dietary complement.

### Carbohydrates

The addition of larvae and its combination with the seed and pseudocereal diminished the proportion of carbohydrates; HTQ ice cream was the exception. This harbored the greatest quantities of carbohydrates even in comparison to HCOo, which is due to the fact that quinoa has a proportion of starch of 53.1% (54).

### Reducing Carbohydrates

In dry basis analyses, it was observed that only the HT formulation had significant differences compared to the control, as these sugars found in the ice cream derive from strawberries, cranberries, and milk.

### Dietary Fiber

As Table 1 shows, only the HTC formulation showed a significant difference with respect to the control ice cream, which is due to the fact that the seed provides this component and in doing so its consumption favors good health and prevents diseases (55). The ice cream we manufactured provided 15–25% of the RDI (Recommended Daily Ingestion) for men and 16–30% for women. That is, consuming 100 g of ice cream covers an important quantity of required fiber per day.

In Table 3, we present a comparative analysis of some foods prepared with insects and examples of foods made with insects. It can be observed that *Bunaeopsis aurantiaca* and *Imbrasia oyemensis* are rich in calories; *B. aurantiaca, I. oyemensis*, and *Rhynchophorus phoenicis* have similar protein amounts to beef; *Cirina forda*, wheat flour, and the different formulations of ice cream are high in nitrogen-free extract, and with respect to ash, the highest amount corresponds to *C. forda*.

**Table 3:** Comparative analysis of the nutritional value of ice cream with other foods made from insects and beef at a g/100 g dry basis.

**Scientific name**	**Prepared as**	**Energy (kcal/100 g)**	**Protein (g)**	**Fat (g)**	**Nitrogen-free extract (g)**	**Ash (g)**	**R**
*Bunaeopsis aurantiaca*	Flour^1^	433.00	24.20	49.00	4.50	3.20	a
*Imbrasia oyemensis*	Flour^2^	477.00	23.70	57.70	11.01	2.60	a
*Cirina forda*	Flour^3^	410.00	12.50	20.00	54.30	8.70	a
*Rhynchophorus phoenicis*	Cookies^4^	477	25.06	26.47	34.83	3.08	b
Wheat flour		474.98	10.89	19.82	63.26	2.54	b
HCOo	Ice cream	388.07	9.36	11.07	62.75	1.8	
HT	Ice cream	407.66	11.81	14.34	57.84	2.11	
HTC	Ice cream	383.75	11.36	15.55	49.59	2.24	
HTQ	Ice cream	389.13	10.90	12.01	59.36	2.02	
Beef		277.00	25.60	18.70			c

*a, Ombeni and Munyuli (56); b, Adedayo et al. (57); c, Schockley and Dossey (58); R, Reference*;

1 * = contains sorghum flour, maize flour, liquid oil, and sugar*;

2 * = contains maize flour, liquid oil, and sugar*;

3 *= contains sorghum flour maize flour, oil, sugar, and salt; and*

4 * = contains 50% wheat flour and 50% R. phoenicis*.

It is especially observed that cookies made with *R. phoenicis* and wheat flour are enriched in proteins by 130%, since wheat flour only has 10.89. In addition, there are published data that point out that the nutritional value of mealworm is comparable to that of beef (23); therefore, *Tenebrio* is ideal to enrich various types of conventional foods such as ice cream. Furthermore, the possibility of using *Tenebrio* larvae as commercial food has been demonstrated as well as evaluated to enrich various foods since they provide a high amount of trace elements, fatty acids, and vitamins.

We must emphasize the nutritional aspects, because this is an important reason for eating insects, and according to numerous edible insect publications around the world, we know that enriching foods high in carbohydrates with proteins of insect origin with a quality comparable to those of chicken, pork, or beef will be a success in the future, as in Tokyo, where they already sell them in special dispensers, and so we must focus on their production without destroying ecosystems. In the case of Mexico, numerous industries have been created, among which the following stand out: Abeja reina, Acetta, Becrikets, Bicho, Brinkos, Criks, Engrillo, Fertifrass, Gran Mitla, Gricha, In insect nutrition, Okuilli, Once chance, Optiprotein, Packs, Q-Kis, Sal de aquí, Santa colmena, Smart bites, Tanti, Totolines, and Zofo, which currently commercialize various products made from insects such as snacks, flours, salts, sauces, fertilizers, “totolines,” and “mescal.” For example, the company called Okuilli markets *T. molitor* larvae flour at a price of $1,150.00 per kilo, which at the exchange rate corresponds to 54 American dollars; this indicates that this activity is a business in continuous growth, both in our country and abroad, whose net earnings are significant. And in Europe, the world's largest producer of *Tenebrio* is called Ynsect. This is a French company located in Dole, near Dijon in eastern France, which breeds and processes yellow mealworms, transforming them into proteins, fats, and chitin that are used in feeding fish and domestic animals and as fertilizers.

Products made from insects also offer great expectations for the pharmaceutical, chemical, and food industries.

### Minerals

Table 4 shows the mineral profile (expressed as mg/100 g of dry sample) of the manufactured formulations.

**Table 4:** Profiles of minerals and vitamins quantified in the manufactured formulations.

**Minerals (mg/100 g of dry sample)**				
	**HCO**	**HT**	**HTC**	**HTQ**
Calcium	254.70^*^ ± 1.11	265.42^b^ ± 0.60	332.04^**^ ± 2.77	239.05^b^ ± 1.58
Phosphorus	156.36^a^ ± 4.39	185.07^b^ ± 4.34	**224.50**^**b**^ ± 0.34	198.98^b^ ± 2.06
Sodium	202.04^a^ ± 8.14	215.27^a^ ± 3.14	**232.78**^**b**^ ± 6.37	188.66^a^ ± 4.99
Potassium	337.93^a^ ± 8.43	403.97^b^ ± 1.57	**408.21**^**b**^ ± 7.35	365.43^b^ ± 6.99
Magnesium	23.92^a^ ± 0.83	32.11^b^ ± 0.89	**50.01**^**b**^ ± 0.58	42.07^b^ ± 0.77
Iron	1.05^a^ ± 0.07	1.15^a^ ± 0.07	1.58^b^ ± 0.06	2.23^b^ ± 0.12
**Liposoluble vitamins (mg/100 g of dry sample)**				
A, retinol	0.97^a^ ± 0.01	1.04^b^ ± 0.01	**1.89**^**b**^ ± 0.00	1.50^b^ ± 0.00
D, calciferol (μg/100 g)	479.74^a^ ± 8.87	584.34^b^ ± 11.5	**949.23**^**b**^ ± 9.77	679.30^b^ ± 14.9
E, α-tocopherol	4.87^a^ ± 0.09	5.93^b^ ± 0.08	**9.73**^**b**^ ± 0.14	6.34^b^ ± 0.10
K, phylloquinone (μg/100 g)	N/D	N/D	**997.0**^**b**^ ± 18.55	554.75^b^ ± 24.3
**Hydrosoluble vitamins (mg/100 g of dry sample)**				
Thiamine, B1	21.53^a^ ± 0.20	22.60^b^ ± 0.61	**27.34**^**b**^ ± 0.31	25.03^b^ ± 0.12
Riboflavin, B2	30.58^a^ ± 0.14	33.74^b^ ± 0.02	33.35^b^ ± 0.32	**35.02**^**b**^ ± 0.32
Niacin, B3	74.55^a^ ± 1.07	86.69^b^ ± 0.42	**102.95**^**b**^ ± 0.58	90.10^b^ ± 0.92
Folic acid, B9	17.50^a^ ± 0.1	36.82^b^ ± 0.58	**39.97**^**b**^ ± 0.41	38.73^b^ ± 0.58
Cobalamin, B12	4.35^a^ ± 0.02	**4.39**^**a**^ ± 0.02	4.26^b^ ± 0.01	4.17^b^ ± 0.01

*HCOo, control ice cream; HT, Tenebrio molitor ice cream; HTC, Tenebrio and chia ice cream; HTQ, Tenebrio and quinoa ice cream; N/D, not determined*.

* *The values in the rows with a different letter indicate that there exists a significant difference among them. Coefficient of variation (CV), known as relative standard deviation (RSD), corresponds to the average value of the three samples processed, that is, <3% and with an alpha level of significance equal to 0.05 (α = 0.05). The highest values quantified in each determination made in bold*.

### Calcium (Ca)

As can be observed, all of the formulations have significant differences in concentrations, and comparatively, HTQ showed a decrease of this mineral. In this respect, Vilcacundo and Hernández-Ledesma (59) pointed out that quinoa has a high calcium concentration, but during the “popping” process which it was submitted to, it could have lost calcium. The inclusion of chia in the HTC ice cream had positive effects as it increased its calcium content; this is understandable as this seed contains six times more calcium than milk (60). The experimental HTQ and HTC ice creams only present 239.058–332.048 mg of calcium; that is, none surpasses the 90 mg of the mineral indicated by the NOM-051-SCFI/SSA1-2010 (47). This is why it is suggested that the diet must include other foodstuffs rich in such a mineral to comply with this requirement.

### Phosphorus (P)

As shown in Table 4, with the addition of larvae, chia, and quinoa, the experimental ice cream had a significant increase in its phosphorus concentration. The highest values were present in the HTC and HTQ formulations since phosphorus is the second most abundant mineral (706 mg/100 g) in their composition. The individual inclusion of larvae within the ice cream had a favorable response, but in the combinations larvae/seeds and larvae/pseudocereal, the concentrations found were higher because the combination of two ingredients high in phosphorus renders a foodstuff that is rich in such a mineral. Nevertheless, even with an increase of phosphorus in the product, the consumption of a 100-g portion provides only 9–12% of the RDI according to the NOM-051-SCFI/SSA1-2010. This is why we recommend the consumption of this product in the diet only as a phosphorus complement.

### Sodium (Na)

In the HTC formulation, a significant difference can be observed as compared to the control ice cream. Even when the literature mentions that the seed's sodium content is very low (61), when added to the product, a significant increase is observed, and this can be explained by the variability of the seed composition that depends on the type of soil and climate it is cultivated in. With the aim of reducing cardiovascular disease, the WHO (62) recommends lowering sodium consumption: for adults, it suggests a daily intake below 2% (5 g of salt). As Table 4 shows, no ice cream surpasses the daily intake limits established by this association.

### Potassium (K)

The addition of larvae, chia, and quinoa significantly increased the potassium content in all of the formulations. HTC is, among all of the formulations, the ice cream that provides the highest quantity of this mineral; to this respect, Muñoz et al. (60) pointed out that potassium is the third most abundant microelement in the seed (407 mg/100 g). On the other hand, even when the HTQ formulation presented no evident potassium increase, the important fact is that such a mineral is present in a bioavailable form (59); therefore it is considered essential in the diet. For this mineral, the WHO (63) recommends a daily intake of 351 mg; since our ice cream only provides 3–5% of this quantity, the consumption of foodstuffs rich in this mineral is recommended to meet this need.

### Magnesium (Mg)

Magnesium is found in a very low concentration in the control ice cream HCOo. Nevertheless, the addition of larvae, chia, and quinoa significantly increased this mineral's levels as compared to HCOo. In the HTC ice cream, chia was the ingredient that significantly provided the highest quantity of Mg, since this seed has a rough content of 335 mg/100 g. The NOM-051-SCFId/SSA1-2010 points out that the RDI for magnesium is 248 mg; our ice cream provides only 3–8% of this mineral, which is why they are recommended only as an alimentary complement.

### Iron (Fe)

Iron was the mineral quantified in the lowest concentration in all of the ice creams; it is found in trace quantities, and because of this, its levels were minimal. It was observed that just with the addition of larvae, there was no difference with respect to HCOo, but in HTC and HTQ, a significant increase was found. This is due, for example, to chia having a high content of this mineral (7.7 mg/100 g). HTQ ice cream was the one that had the highest Fe concentration; this is because quinoa has 1,416.7 mg Fe/100 g upon a dry basis. However, its content does not reach a whole milligram in the final product; that is, ice cream provides 25% of the RDI according to NOM-051-SCFI/SSA1-2010, 17 mg. Therefore, we recommend the consumption of iron-rich foodstuffs to comply with this requirement.

### Liposoluble Vitamins

Table 4 shows the results obtained in relation to the levels of liposoluble vitamins in the formulations upon a dry basis.

### Vitamin A (Retinol)

In this case, it was observed that all of the formulations had an increase due to the addition of the ingredients. Nonetheless, the highest value was found in the HTC formulation, due to the fact that this vitamin is the most abundant in the seed with 44 μg/100 g (64). That is, the combination larvae/seed achieved a synergic effect since larvae contain this vitamin but in a lower quantity than that in chia, which has 29 UI/100 g. A significant increase has been shown by the HTQ ice cream; it is minimal when compared to HTC, but better than HCOo.

According to the FAO (49), quinoa is composed of retinol in a range of 0.12–0.53 mg/100 g. However, before the addition of quinoa to the ice cream, it was submitted to two thermal processes, “popping” and drying, which could have had an effect on the vitamin's stability, causing its oxidation. The NOM-051-SCFI/SSA1-2010 points out that the RDI for vitamin A is 568 μg; as observed in Table 4, the HTC product complies with such a requirement, compared to the HCOo, HT, and HTQ formulations, which only provided 66–69% of retinol.

### Vitamin D (Calciferol)

In this research, the highest concentrations were found in the ice cream to which chia had been added (HTC). Nevertheless, for the ice cream added with quinoa, a high content of vitamin D was also obtained. The NOM-051-SCFI/SSA1-2010 indicates an RDI of 5.6 μg; as can be seen, all of the ice creams surpassed this figure, but the occasional consumption of these ice creams would not affect consumers' health.

### Vitamin E (α-Tocopherol)

It was quantified in a high proportion in the formulations, as they showed a higher quantity than the control ice cream. Nonetheless, when only larvae were added, the increase of vitamin E was minimal, which is due to the fact that larvae have very low concentrations of this vitamin (1.9 mg/100 g) as compared to chia and quinoa (65). On the other hand, when chia was added, we saw a higher increase of vitamin E in HTC, because of the large amount of this vitamin in chia (23,842.7 mg/100 g) (60).

Quinoa is also an abundant source of vitamin E, whose concentration ranges from 3.7 to 6.0 mg/100 g; however, when the pseudocereal is mixed with the larvae, vitamin E concentration diminishes because—as both ingredients have low levels of tocopherols—the combination of both dilutes them. The NOM-051-SCFI/SSA1-2010 points out that according to the RDI of vitamin E, one must consume 11 mg of it. As Table 4 shows, our ice cream provides 9–36% of that vitamin, and therefore, the consumption of complementary foodstuffs with tocopherol content is recommended.

### Vitamin K (Phylloquinone)

In Table 4, it is observed that the quantity of vitamin K that HTC and HTQ provide is due to the addition of chia and quinoa and that comparatively the larvae provide a minimal proportion, given that when HT was analyzed, its concentration could not be quantified. NOM-051-SCFI/SSA1-2010 indicates an RDI of 78 μg. As Table 4 shows, the ice cream in which this vitamin was quantified complies with such a requirement, but—since these products are recommended as dietary complements—excess consumption does not pose any health risks.

### Hydrosoluble Vitamins

In Table 4, we present the concentration of hydrosoluble vitamins found in the formulations upon a sample of mg/100 g dry basis.

### Thiamine (B1)

All of the experimental formulations showed a significant increase. However, HTC is the one that provides the highest quantity of this vitamin, because, as Muñoz et al. (60) have reported, chia has an important thiamine content (0.62 mg/100 g). We must point out that even when larvae were the ones that contributed less to this enrichment, they acted in synergy with the chia seed. On the other hand, the addition of quinoa also favored a thiamine increase in the ice cream of up to 15%, which is due to the fact that quinoa harbors a concentration of 0.4 mg/100 g of vitamin B1 (66). All of the ice creams comply with the RDI established by the NOM-051-SCFI/SSA1-2010: 800 μg; that is, the consumption of a portion of 100 g of any of them will meet the daily requirement of this vitamin.

### Riboflavin (B2)

The results in Table 4 show that a significant increase occurred in all of the formulations. In the same way, it was observed that larvae are the ones that provide the highest quantity of B2 in the products, and when combined with quinoa, the formulation's enrichment is even higher. This is due to the fact that the larvae have 1.21 mg/100 g of riboflavin, and quinoa has 0.39 mg/100 g (65, 66), so that, when the two ingredients are combined, a synergy occurs resulting in an increase of vitamin B2 of up to 14% as compared to the control formulation. Meanwhile, in the HTC sample, we quantified a lower level, since chia only provides 0.17 mg/100 g of this vitamin.

### Niacin (B3)

Niacin was the vitamin that had the highest concentrations in the ice cream, even in the control formulation, since milk contains tryptophan—which is considered niacin's precursor—in a significant quantity: 7 mg/g protein. According to Table 4, when larvae and the seed were mixed in the HTC, this content was reinforced up to 38%, given that chia is considered a rich source of this vitamin with a content of 8.83 mg/100 g (60), and larvae contain 4.10 mg/100 g. In such a way, when combined, they present a synergy, with a product rich in niacin being the result. When the larvae and the pseudocereal were combined, the increase was minimal, but still higher than the quantity found in HCOo.

### Folic Acid (B9)

In all of the experimental formulations, significant increases were obtained when compared with HCOo. The fortification of this vitamin in the ice creams can be appreciated, as when an extra ingredient or their combination is incorporated, an increase of up to 87% is observed. As Table 4 shows, larvae provide the highest quantity of vitamin B9, because, as Novak et al. (65) have mentioned, these possess a concentration of 137 mg/100 g. In the same way, it can be observed that when larvae are combined with the seed, the amount of folic acid increases up to 128% with respect to the control ice cream.

### Cobalamin (B12)

Table 4 shows that cobalamin was the vitamin quantified in the lowest concentration in the ice cream, HT being the one that had the highest quantity. On the other hand, when larvae were included in HTC and HTQ, since its content is minimal (0.30 μg 100 g^−1^), there was a decrease as compared to that of the HCOo; that is, the larvae favored only a dilution of this vitamin in the formulations. Unfortunately, there are no data about the quantity of this vitamin in these ingredients that would enable us to draw a quantitative comparison. Notwithstanding this situation, the quantities of B12 in the ice creams effectively cover the RDI of 2.1 μg (0.0021 mg) that the NOM-051-SCFI/SSA1-2010 recommends.

### Anthocyanins

**Figure 1:**
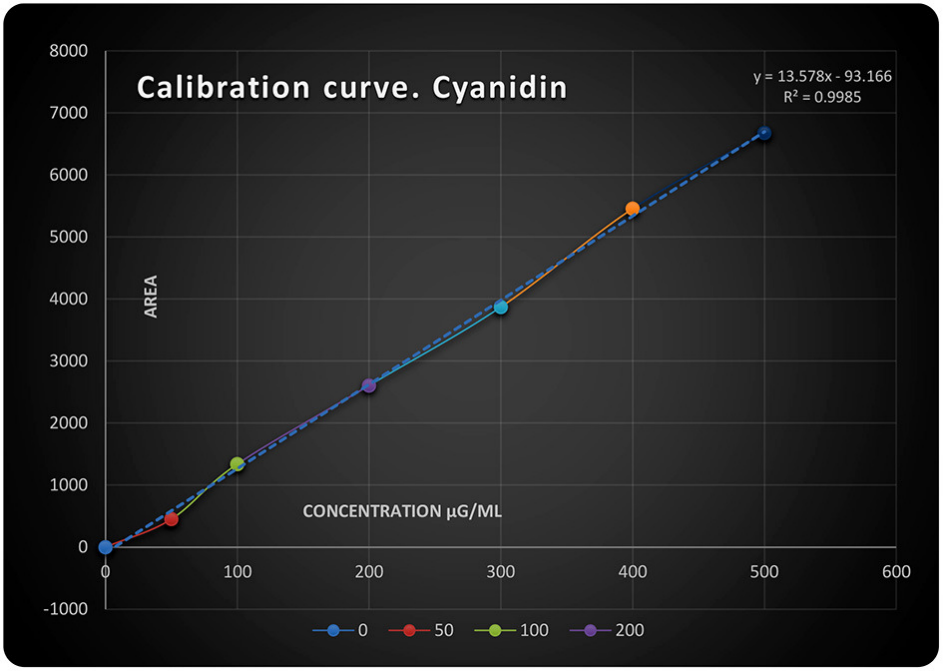
Cyanidin chloride calibration curve.

In Tables 5, 6 and Figure 1, we present the concentrations of cyanidin chloride quantified in the different ice creams upon a dry basis. The content of cyanidin in the four formulations derives from the strawberries and cranberries that give the ice cream their flavor. The table shows that there were significant differences as compared to HCOo. The formulations with the highest concentrations of anthocyanins were HT and HTC. The latter had the highest levels of anthocyanins, which could be due to the quantity of antioxidants found in the seed, caffeic acid (6.6 mg/100 g), and chlorogenic acid (7.1 mg/100 g), which, in turn, increases cyanidin's stability (64). On the other hand, even when the pseudocereal is an abundant source of antioxidants (66), HTQ had a lower concentration of cyanidin. This is due to the process of “popping” and drying to which quinoa was submitted and which made it lose its antioxidant activity and, thus, its cyanidin concentration, given that it becomes unstable in the presence of oxygen.

**Table 5:** Composition of the four formulations (%).

	**Formulations**			
***Ingredient***	**Control ice cream, HCOo**	***Tenebrio molitor* ice cream, HT**	***Tenebrio* and chia ice cream, HTC**	***Tenebrio* and quinoa ice cream, HTQ**
Ice cream base^*^	40.0	31.5	21.5	21.5
Strawberry	30.0	30.0	30.0	30.0
Cranberry	30.0	30.0	30.0	30.0
*Tenebrio molitor* larvae	–	8.5	8.5	8.5
Chia	–	–	10.0	–
Quinoa	–	–	–	10.0

* *The components of the control ice cream are the following: water, powdered whole milk, sugar, carob bean gum, carboxymethyl cellulose (CMC), carrageenan gum, strawberry, and cranberry in 1 kg*.

**Table 6:** Cyanidin chloride concentration upon a dry basis of the formulations.

**Sample**	**Concentration (mg/100 g of dry sample)**
Control ice cream HCOo	33.39^a^ ± 0.00
*Tenebrio* ice cream HT	36.04^b^ ± 0.00
*Tenebrio* chia ice cream HTC	42.35^b^ ± 0.00
*Tenebrio* quinoa ice cream HTQ	31.57^b^ ± 0.00

*HCOo, control ice cream; HT, Tenebrio molitor ice cream; HTC, Tenebrio and chia ice cream; and HTQ, Tenebrio and quinoa ice cream. *The values in the rows with a different letter indicate that there exists a significant difference among them. Coefficient of variation (CV), known as relative standard deviation (RSD), corresponds to the average value of the three samples processed, that is, <3% and with an alpha level of significance equal to 0.05 (α = 0.05).

### Strengths and Weaknesses

In this case, the nutritional value of the ice creams prepared with insect addition was improved in macronutrients and micronutrients, as compared to the control batch. Therefore, in this research, we focus on the potential application of ice creams with diverse functional properties.

The results shown with the experimental batches of ice cream are an important contribution in the elaboration of nutritious and functional foods in the future (67).

Besides, as the yellow mealworm is an organism with a short life cycle whose cultivation is simple, it has been developed on an industrial scale, for example, in Europe. It is also widely known that various foods prepared with the larvae of the yellow mealworm are consumed directly after their culinary preparation; for example, technological innovations include the development of nutritional bars and bread, and diverse high-quality foodstuffs can be obtained to meet various needs of both man and pet animals such as dogs and cats, being also beneficial to the growth and development of poultry and livestock. It is generally believed that it can provide a good deal of comprehensive and rich nutrients (68) and can also be made into common and processed foods. Predictably, new deep processed foods, such as jam, beverages, vinegar, and wine, along with powder, capsules, and tablets, will be available in the market, as has been done with *Clanis bilineata tsingtauica* (67).

Moreover, due to the antioxidant and anti-aging activity of the larvae, corresponding components can be extracted to develop more functional foods and could be a suitable functional food resource for humans due to its composition of active substances.

However, further research is required to assess the effects of preparation on foodstuffs when the larvae of this beetle are added; it is necessary to carry out statistical sensory analyses, compare shelf life characteristics, and, in the case of ice creams, prepare them according to the dietary needs of the people who consume them, for example, diabetics, considering their age, weight, gender, height, occupational activity, etc. It would also be necessary to establish a business plan and increase production on a larger scale to be able to commercialize the ice creams at the national and international markets.

Besides, to know the nutritional value of some edible insects and several conventional foods, statistical models called Ofcom and the Nutrient Value Score (NVS) have been used, as well as non-parametric tests and Bonferroni adjustments to verify nutritional differences. Of the available insects studied, the ones that are commercially important are *Bombyx mori, Imbrasia belina, R. phoenicis* (Fabricius, 1801), *T. molitor* Linnaeus, 1758, *Acheta domesticu*s (Linnaeus, 1758), and *Apis mellifera*; they were compared with beef, pork, and chicken. Details of the methods are found in Payne et al. (69).

According to the Ofcom model, insects are not healthier than meat products, and with the results of the NVS method, crickets and yellow worms are significantly healthier than beef and chicken.

In this publication, it is reported that crickets and bees are richer in iron compared to beef, pork, and chicken and higher in calcium and riboflavin than meat and meat offal, but there is no indication that the insects investigated are statistically healthier than meat. However, their results are not conclusive at this time and should therefore be treated with reserve.

In any case, the nutritional quality of the insects studied is “relatively” unimportant since in the daily diet through the use of mixed diets, the phenomenon of complementation/supplementation between different proteins occurs. Food supplementation is a concept used to design diets or foods in which different sources of protein are mixed in order to improve the quality of the resulting combination.

## Conclusions

The three formulations—HT, HTC, and HTQ—showed a noteworthy augmentation in the previously examined macronutrients and micronutrients in comparison to the control formulation. After a thorough examination, it could be observed that the HTC formulation has proven to be the best one due to containing the upmost values of essential nutrients such as protein, fat, dietary fiber, ash, minerals, cyanidin, and vitamins. According to the formulations of the ice creams made, the amount of protein increased by 41%; that is, these larvae are ideal to enrich various types of conventional foods such as ice cream, and this will be a success in the future. Likewise, ice creams improved significantly in fatty acids, vitamins (A, E, B2, B3, and B9), and minerals (phosphorus, potassium, magnesium, and antioxidants); therefore, the larvae have a high potential to supplement conventional foods.

To sum up, we can conclude by saying that the importance of creating new formulations of functional food ice cream lies not only in its nutritional value but also in the functional macronutrients and micronutrients, which, all in all, effectively prevent any sort of disease.

For the aforementioned reasons, the food industry is looking for alternative nutritional sources such as insects, for the elaboration of products; such is the case of the nutraceutical ice creams analyzed and the protein enrichment of various conventional foods, among which we have “jungle bars,” cookies, tortillas, cakes, chocolates, and sausages. At present, various foods made with *T. molitor* larvae such as ice cream are a novel and emerging food industry. Therefore, it may be developed as a sustainable source of alternative resources for human consumption and widely used in many other investigations such as animal feeding and the elaboration of some functional foods.

The benefits of using insects as human food are diverse; moreover, based upon this premise, we can claim that insects have a high potential of becoming a new sector in the constantly innovative food industry of Mexico.

## Data Availability Statement

The raw data supporting the conclusions of this article will be made available by the authors, without undue reservation.

## Author Contributions

All authors listed have made a substantial, direct, and intellectual contribution to the work and approved it for publication.

## Conflict of Interest

The authors declare that the research was conducted in the absence of any commercial or financial relationships that could be construed as a potential conflict of interest.

## Publisher's Note

All claims expressed in this article are solely those of the authors and do not necessarily represent those of their affiliated organizations, or those of the publisher, the editors and the reviewers. Any product that may be evaluated in this article, or claim that may be made by its manufacturer, is not guaranteed or endorsed by the publisher.
