# Supplementary material for: Development of Nutraceutical Ice Creams Using Flour Yellow Worm Larvae (Tenebrio molitor), Chia (Salvia hispanica), and Quinoa (Chenopodium quinoa)
Source: Front Vet Sci. 2021 Dec 7;8:629180. doi: 10.3389/fvets.2021.629180 (PMC8688395; doi:10.3389/fvets.2021.629180)
Supplement: Supplementary file 1 [file Data_Sheet_1.docx]

**Supplementary information**

**Fatty acids**


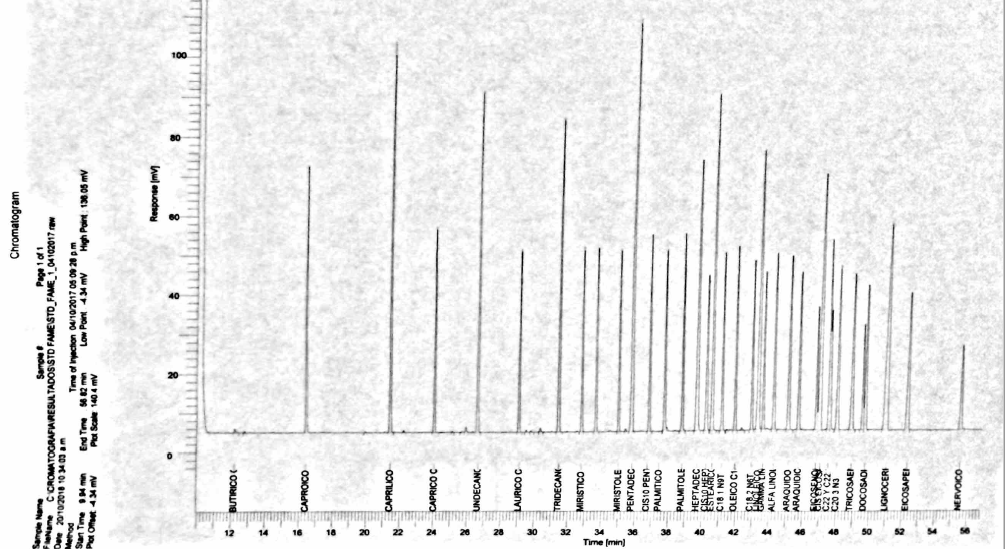


**1 Chromatogram of the Supelco standard for quantification of fatty acids.**

| Fatty acids | Holding time |
| --- | --- |
| **Butyric C4** | 12.16 |
| **Caproic C6** | 12.72 |
| **Caprylic C8** | 16.9 |
| **Capric C10** | 21.41 |
| **Undecanoic C11** | 22.18 |
| **Lauric C12** | 24.02 |
| **Tridecanoic C13** | 25.1 |
| **Myristic C14** | 27.75 |
| **Myristoleic C14:1** | 26.32 |
| **Pentadecanoic C15** | 26.63 |
| **Cis-10 Pentadecanoic** | 29.06 |
| **Palmitic C16** | 29.48 |
| **Palmitoleic C16:1** | 29.76 |
| **Heptadecanoic** | 30.01 |
| **cis-10 Heptadecanoic** | 31.43 |
| **Stearic C18** | 32.52 |
| **C18:1 N9T** | 32.79 |
| **Oleic C18:1 N6C** | 33.62 |
| **C18:2 N6T** | 34.98 |
| **Linoleic C18:2 N6C** | 35.37 |
| **Gamma linolenic C18:3** | 35.79 |
| **Alpha linolenic C18:3** | 36.8 |
| **Arachidic C20** | 37.72 |
| **Eicosenoic C20:1** | 38.77 |
| **Heneicosenoic C21** | 39.66 |
| **Cis eicosadienoic C20:2** | 40.2 |
| **C22 Y C22:1** | 40.55 |
| **C20:3 N3** | 41.13 |
| **Tricaseenozium C23** | 41.4 |
| **Arachidonic C20:4** | 41.91 |
| **Docosadienoic C22:2** | 42.29 |
| **Lignoceric C24** | 42.89 |
| **Eicosapentaenoic C20:5** | 43.29 |
| **Nervoic C24:1** | 43.57 |
| **Docosahexaenoic C22:6** | 44.2 |

**2 Retention time results for fatty acids.**


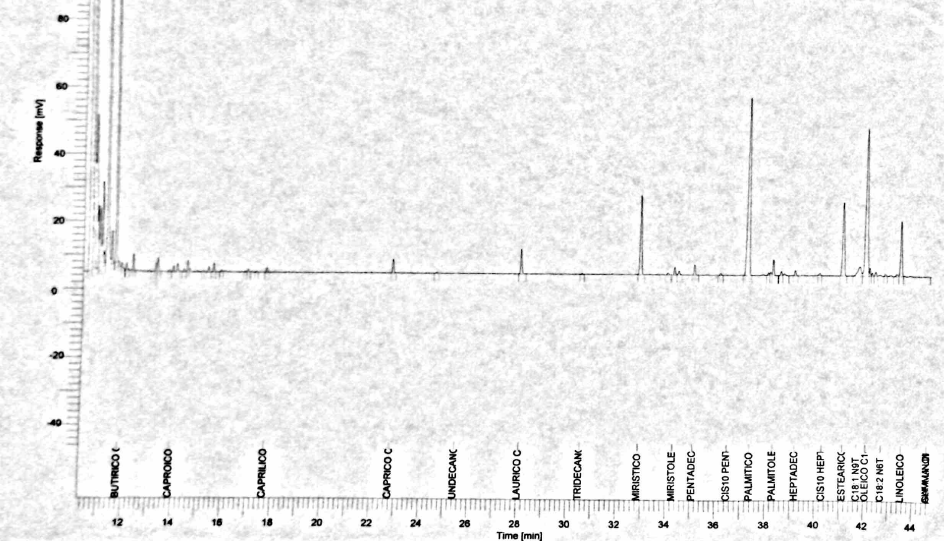


**3 Chromatogram of the fatty acid profile for the *Tenebrio* chia formulation.**

| **Vitamin A λ=275 nm** | | |
| --- | --- | --- |
| **Tr** | µg/mL | Area |
| **5.144** | 500 | 1220.677 |
| **5.104** | 400 | 998.455 |
| **5.147** | 300 | 777.564 |
| **5.222** | 200 | 518.469 |
| **5.147** | 100 | 248.032 |
| **--------** | 0 | --------- |

**4 Results of the vitamin A calibration curve**

**5 Vitamin A calibration curve**

| Vitamin D λ=275 nm | | |
| --- | --- | --- |
| Tr | µg/mL | Area |
| 14.952 | 500 | 2764.694 |
| 14.638 | 400 | 2264.826 |
| 14.886 | 300 | 1765.49 |
| 15.236 | 200 | 1081.433 |
| 14.9755 | 100 | 570.469 |
| ---------- | 0 | ---------- |

**6 Results of the vitamin D calibration curve**

**7 Vitamin D calibration curve**

| Vitamin K λ=275 nm | | |
| --- | --- | --- |
| TR | µg/mL | Area |
| 28.886 | 500 | 1533.378 |
| 28.152 | 400 | 1255.4 |
| 28.774 | 300 | 973.098 |
| 29.103 | 200 | 654.269 |
| 29.126 | 100 | 312.677 |
| ------ | 0 | ------- |

**8 Results of the vitamin K calibration curve**

**9 Vitamin K calibration curve**

| Vitamin E λ=285 | | |
| --- | --- | --- |
| Tr | µg/mL | Área |
| 18.807 | 1000 | 515.312 |
| 18.389 | 600 | 311.295 |
| 18.739 | 400 | 221.334 |
| 19.15 | 200 | 104.346 |
| -------- | 0 | --------- |

**10 Results of the vitamin E calibration curve**

**11 Vitamin E calibration curve**

**12 Chromatogram of the stock profile of fat-soluble vitamins λ 275**

**13 Chromatogram of the fat-soluble vitamin stock profile λ 285**

**14 Chromatogram of the fat-soluble vitamin profile of the formulation HTC λ 285**

**15 Chromatogram of the profile of fat-soluble vitamins in the formulation HTC λ 275**

| Tr | µg/mL | Area |
| --- | --- | --- |
| 1.85 | 500 | 1787424.17 |
| 1.813 | 400 | 1186934.17 |
| 1.815 | 300 | 860819.167 |
| 1.807 | 200 | 604070 |
| 1.8 | 100 | 281238.75 |
| 1.786 | 50 | 109879.167 |
| ------- | 0 | ------- |

**16 Results of the calibration curve for vitamin B1**

**17 Vitamin B1 calibration curve**

| Tr | µg/mL | Area |
| --- | --- | --- |
| 8.016 | 500 | 4134405.83 |
| 8.031 | 400 | 3212082.5 |
| 8.055 | 300 | 2351280 |
| 8.025 | 200 | 1609538.33 |
| 8.047 | 100 | 340743.75 |
| 8.094 | 50 | 156636.667 |
| ------- | 0 | -------- |

**18 Results of the calibration curve for vitamin B2**

**19 Calibration curve for vitamin B2**

| Tr | µg/mL | Area |
| --- | --- | --- |
| 5.125 | 500 | 2144949.24 |
| 5.074 | 400 | 1663645.83 |
| 5.085 | 300 | 1242775 |
| 5.069 | 200 | 825205.833 |
| 5.048 | 100 | 409283.75 |
| 5.009 | 50 | 84842.5 |
| ------- | 0 | --------- |

**20 Results of the vitamin B3 calibration curve**

**21 Calibration curve for vitamin B3**

| Tr | µg/mL | Area |
| --- | --- | --- |
| 4.646 | 500 | 330243.33 |
| 4.604 | 400 | 252498.333 |
| 4.639 | 300 | 182152.5 |
| 4.649 | 200 | 114375.833 |
| 4.637 | 100 | 56140.833 |
| 4.609 | 50 | 23995.417 |
| ------- | 0 | ---------- |

**22 Results of the calibration curve for vitamin B6**

**23 Calibration curve for vitamin B6**

| Tr | µg/mL | Area |
| --- | --- | --- |
| 6.918 | 500 | 2014938.69 |
| 6.923 | 400 | 1554450.87 |
| 6.929 | 300 | 1156554.58 |
| 6.892 | 200 | 793414.281 |
| 6.913 | 100 | 419583.313 |
| 6.928 | 50 | 162989.167 |
| ------ | 0 | -------- |

**24 Results of the calibration curve for vitamin B9**

**25 Calibration curve for vitamin B9**

| Tr | µg/mL | Area |
| --- | --- | --- |
| 7.373 | 500 | 2560918.74 |
| 7.381 | 400 | 1976370 |
| 7.394 | 300 | 1454547.5 |
| 7.349 | 200 | 995345 |
| 7.372 | 100 | 504258.333 |
| 7.416 | 50 | 239133.333 |
| ----- | 0 | ------- |

**26 Results of the calibration curve for vitamin B12**

**27 Calibration curve for vitamin B12**


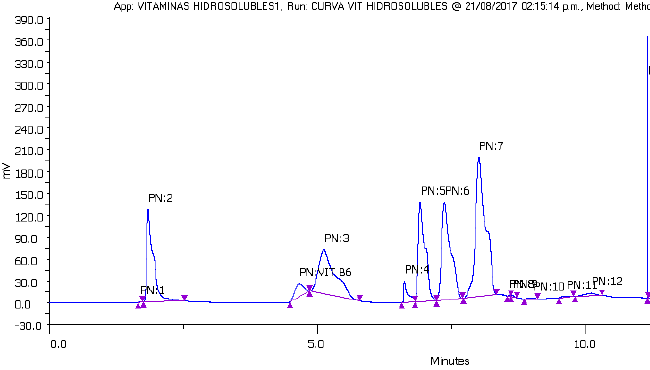
**28 Chromatogram of the stock profile of water-soluble vitamins**


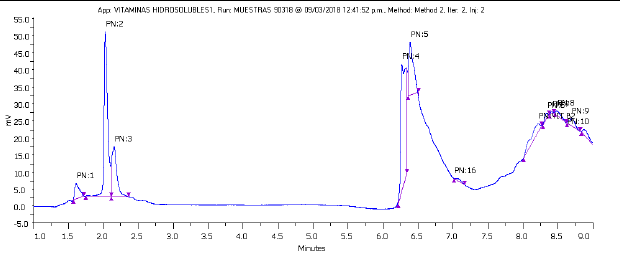
**29 Chromatogram of the water-soluble vitamin profile in ice cream added with quinoa**

| Concentration µg/ml | Area |
| --- | --- |
| 0 | 0 |
| 50 | 451.408 |
| 100 | 1338.815 |
| 200 | 2604.867 |
| 300 | 3869.596 |
| 400 | 5454.84 |
| 500 | 6674.854 |

**30 Results of the calibration curve for cyanidin chloride**

**31 Cyanidin Chloride Calibration Curve**

**32 Chromatogram of cyanidin chloride**

**33 Chromatogram of the analysis of cyanidin chloride in the HC formulation**
